# News and Coming Events

**Published:** 1908-02-29

**Authors:** 


### NEWS AND COMING EVENTS.

At the Southwark Coroner's Court Dr. F. J. Waldo held an inquest on Edward James Shaw (40), late a horse dealer, who died on February 6 from glanders.

---

Dr. Comyns Berkeley and Dr. Herbert Williamson have been appointed physicians to in-patients to the City of London Lying-in Hospital.

---

The second reading is announced for March 3 of the Bill which Sir J. Batty Tuke has introduced to prohibit joint-stock companies from acting as physicians, surgeons, and medical practitioners. The Bill has the same form as it had when it left the House of Lords last session.

---

The directors of the Royal Asylum Morningside, Edinburgh, on February 20, appointed Dr. George M. Robertson, superintendent of Stirling District Asylum, Larbert, to be successor to Dr. T. S. Clouston, physician-superintendent.

---

At a meeting of the Senate of the University of London held on February 19 the Vice-Chancellor (Sir William Collins, M.P., M.D.) being in the chair, the following were appointed Fellows of University College:—Dr. Archibald Montague Henry Gray, Mr. Philip Maynard Heath, F.R.C.S., and Professor George R. Murray, M.D.

---

At a meeting of the Council of the University of Leeds the resignation of Mr. Bevan Lewis of his lectureship in mental diseases was accepted, and the following appointments were made: Dr. Croft, Lecturer in Gynæcology; Mr. Andrew Hunter, M.B., Demonstrator of Physiology; Mr. R. Veitch Clark, M.B., Honorary Demonstrator in Public Health.

---

The Lord Mayor presided on February 20 at the 157th annual Court of Governors of the City of London Lying-in Hospital. During last year the number of in- and out-patients was 3,448, exceeding that of any previous year in the history of the charity. In the out-patient department 2,772 women were attended at their own homes. The income during last year reached £5,293, but the expenditure was £6,350.

---

The second triennial international conference on the blind will be held in Manchester on July 27, 28, 29, 30, 31, and August 1. In connection with it there will be an international exhibition, from July 24 to August 3 inclusive, of educational and technical appliances, and specimens of work by the blind. The committee invite the co-operation of those interested in the blind to contribute towards this exhibition and the success of the conference. Mr. W. H. Illingworth, of Henshaw's Blind Asylum, Old Trafford, Manchester, is the hon. general secretary.

---

The Prince of Wales, at the suggestion of the Executive Committee of King Edward's Hospital Fund for London, has decided to appoint a committee to inquire into the system prevailing in the London voluntary hospitals with regard to the admission of out-patients. In view of the fact that some portion of the subject of inquiry by such a committee would probably fall within the subjects to be reported upon by the Royal Commission on the Poor Laws, the committee will not be appointed until the Commission has reported.

The newly appointed Royal Commission on Whisky and other Potable Spirits held their first meeting at the Local Government Board offices in Whitehall on Wednesday, February 26.

---

According to a message from Berlin, Mr. Andrew Carnegie has given half a million marks (£25,000) to the Robert Koch fund for the campaign against tuberculosis. The amount collected so far for carrying out research work in connection with the disease amounts to 800,000 marks (£40,000).

---

On February 18 a general Court of Governors of the Westminster Hospital was held for the election of committees and other annual business. Sir J. Wolfe Barry occupied the chair, supported by Mr. H. D. Erskine, Sir W. H. Allchin, Dr. de Havilland Hall, Mr. C. Stonham, F.R.C.S., Sir Robert Hudson, and other Governors. During the past year 2,437 in-patients and 21,175 out-patients were relieved. The expenditure amounted to £19,955.

---

The annual general meeting of the "Marie Celeste" Samaritan Society was held at the London Hospital, the Hon. Sydney Holland occupying the chair. The number of patients assisted during the past year was 7,815, being an increase of 507 over 1906. The chairman announced a donation of £10 from the Queen. It was reported that the light treatment, started at the hospital by Her Majesty, has now made such headway in the cure of lupus in this country that the number of patients attending will probably diminish rather than increase.

---

A number of officers of the Royal Army Medical Corps have been selected to fill appointments in the Royal Territorial Medical Corps when it becomes properly organised and established. The following, among others, have been selected for the appointment of adjutants and have been ordered to join the R.A.M.C. depôt at Aldershot to undergo a special course of instruction prior to appointment: Captain R. H. Lloyd, Captain J. M. Sloan, Captain W. A. Woodside, Captain E. P. Connolly, Captain H. S. Roch, Captain E. Bennett, and Captain F. A. Stephens.

---

Dr. Henry Robert Silvester, author of the "Silvester Method" of recovering the apparently drowned, died last week at his residence on Clapham Common, in his 80th year. Educated at King's College, London, he graduated B.A. in 1850, was admitted a member of the Royal College of Surgons, England, in 1853, and proceeded to the M.B. degree, London, in 1854, and to the M.D. in 1855. Dr. Silvester, who formerly practised in Clapham, received the Fothergill Gold Medallion of the value of 50 guineas from the Royal Humane Society in 1883, on their adoption of the "Silvester Method." He was the author of several medical works.

---

The annual general Court of Governors of the German Hospital (Dalston) was held on February 18, Mr. A. J. Allen, the chairman, presiding. Reference was made to the visit of the German Empress to the hospital in November and her donation of 1,000 marks, while the Emperor again contributed £200. The in-patients last year numbered 1,592, the out-patients 26,564. The receipts amounted to £10,977, and the expenditure to £11,694. The new convalescent home at Hitchin—the gift of a few anonymous friends—will be ready for occupation at midsummer. The beds will number 40.

Dr. James Collier has just been appointed physician to in-patients at St. George's Hospital.

Dr. R. A. Chisholm has been appointed assistant physician to the North-Eastern Hospital for Children.

Drs. Kingsley Ackland and Sinclair Thompson have been appointed Honorary Consulting Surgeons to the Bideford Infirmary.

The Middlemore Prize, awarded by the British Medical Association for research work into the causation of and treatment of diseases of the eye, has been awarded to Mr. Simeon Snell, F.R.C.S., of Sheffield.

Dr. H. Campbell Thomson, M.D., F.R.C.P., has been elected Dean of the Medical School of the Middlesex Hospital in the place of Mr. John Murray, F.R.C.S., who has resigned, after holding the office for six years.

The deaths attributed to influenza in London last week numbered 126, the totals for the previous three weeks having been 32, 34, and 84. The general death rate for London was 18.5 per thousand, being .5 less than that for the preceding week.

The following appointments have been made at Bristol University College: Director of the University College public health laboratory, J. M. Fortescue Brickdale, M.D.; honorary demonstrators of anatomy, William H. A. Elliott, M.B., B.S., and Charles B. Gouldon, M.C., F.R.C.S.

We understand that an important meeting will take place in the course of next month at the War Office, when the matrons of the chief London and provincial hospitals will attend in order to discuss schemes of nursing in connection with the new territorial system of the Army.

The Local Government Board has notified the Chelsea Board of Guardians that an inquiry has been directed into the management of the infirmary, consequent on the recent death of an inmate. The investigation will be made by the Board's inspectors, Dr. Downes and Dr. Fuller.

The annual meeting of the New Hospital for Women, Euston Road, will be held at the hospital on Tuesday, March 3, 1908, at 3 p.m., under the chairmanship of the Lady Knightley of Fawsley. The speakers will include Sir John Cockburn, K.C.M.G., and Dr. Charles Mercier.

Four lectures will be delivered at Gresham College, Basinghall Street, E.C., on Monday, March 2, Tuesday, March 3, Thursday, March 5, and Friday, March 6, by Dr. F. M. Sandwith, Gresham Professor of Physic. The first lecture will deal with "The Gresham Professors of Physic," the others with "Tuberculosis." The lectures are free to the public, and begin each evening at six o'clock.

The Child Study Society London, will hold the following lectures and discussions during 1908, in the Parkes Museum, Margaret Street, W., on Thursdays, at 8 p.m.: March 5, "The Education (Administrative Provisions) Act in relation to the inspection and treatment of the teeth." By W. T. Elliott, D.D.S., L.D.S. March 12, "Congenital Aphasia (word blindness and word deafness)." By C. J. Thomas, M.B., B.Sc., D.P.H. March 19, "Anthropometry and physical development" (illustrated by lantern slides). By Frank C. Shrubsall, M.A., M.D., M.R.C.P. March 26, "The downfall of the dogma of formal training." By T. H. Hayward, Ph.D. April 2, "On the mechanism of speech and stammering." By M. Friedeberger, Ph.D.

Dr. R. S. Fancourt Barnes died at Worthing, on February 19, at the age of 59. He was the elder son of the late Robert Barnes, the famous obstetrician. As physician and consulting physician he was associated, among other institutions, with the Royal Maternity Charity, the British Lying-in Hospital, and the Chelsea Hospital for Women.

The death is announced of Professor von Esmarch, the well known German military surgeon. He had a long and distinguished career. He was appointed in 1870 to be General Medical Officer and Consulting Surgeon to the army. He did excellent work in the organisation of the volunteer medical corps in the Franco-Prussian War, and immediately afterwards he was attached as consulting surgeon to the principal military hospital at Berlin. The value of his services to the army was recognised by various distinctions, including his elevation in 1897 to the rank of Privy Councillor. He was author of many valuable communications dealing with military surgery and the conduct of military hospitals.

The following lectures will be delivered at the Royal College of Physicians, Pall Mall East, on Tuesdays and Thursdays, at five o'clock: Milroy lectures—Dr. J. W. H. Eyre, March 5, 10, 12; "Melitensis Septicæmia" ("Malta or Mediterranean Fever"). Goulstonian lectures—Dr. Herbert French, March 17, 19, 24; "The Influence of Pregnancy on certain Medical Diseases, and the Influence of certain Medical Diseases on Pregnancy." Lumleian lectures—Sir James Sawyer, M.D., March 26, 31, April 2; "Points of Practice in Maladies of the Heart." Oliver-Sharpey lectures—Prof. E. A. Schäfer, F.R.S., April 7, 9; "The present position of our knowledge regarding the Supra-renal capsules."

The annual meeting of Queen Charlotte's Hospital was held on February 24, Mr. Frederick W. Hunt presiding in the absence, through illness, of Sir Samuel Scott. The report showed that 1,701 patients had been received into the hospital during the year, and 1,996 had been attended and nursed in their own homes. The expenditure had amounted to £6.451, the total income had been £5,778, and the balance, £573, had had to be met by trenching upon capital. The extension of the Nurses' Home was nearing completion, and the Residential College for Students was also being enlarged so as to provide for sixteen medical students instead of six as at present.

Augustus Q. Tucker, of Herne Hill, plaintiff in the recent libel action against the *Lancet*, was summoned before Mr. Francis at the Lambeth Police Court on February 24, for having sold to George Waldock cocaine and atropine, contained in a bottle not distinctly labelled with the word "Poison" or with the name and address of the seller. Mr. W. S. Glyn Jones appeared on behalf of the Pharmaceutical Society, and the defendant was represented by Mr. F. E. Smith, K.C. Mr. Waldock said he obtained a bottle of the specific by instruction from the Pharmaceutical Society. Mr. Thomas Tickle, public analyst for Exeter, spoke to the bottle containing cocaine and atropine. Dr. Peter Daniel, of Harley Street, said that if the contents of the bottle were swallowed they would probably lead to death. The danger would arise from leaving such a bottle without any label or any directions whatever. Mr. Smith said that the defendant had no objection to putting a poison label on the bottles in the future if the Court was of opinion that that should be done. Mr. Francis thought that there had been a breach of the Act, and ordered the defendant to pay a penalty of £5 and £5 5s. costs.

